# Presence in the Pre-Surgical Fine-Needle Aspiration of Potential Thyroid Biomarkers Previously Identified in the Post-Surgical One

**DOI:** 10.1371/journal.pone.0072911

**Published:** 2013-09-02

**Authors:** Federica Ciregia, Laura Giusti, Angelo Molinaro, Filippo Niccolai, Patrizia Agretti, Teresa Rago, Giancarlo Di Coscio, Paolo Vitti, Fulvio Basolo, Pietro Iacconi, Massimo Tonacchera, Antonio Lucacchini

**Affiliations:** 1 Department of Pharmacy, University of Pisa, Pisa, Italy; 2 Department of Clinical and Experimental Medicine, University of Pisa, Pisa, Italy; 3 Section of Cytopathology, University of Pisa and Pisa University Hospital, Pisa, Italy; 4 Department of Surgical, Medical, Molecular Pathology and Critical Area, University of Pisa, Pisa, Italy; Consiglio Nazionale delle Ricerche (CNR), Italy

## Abstract

Fine-needle aspiration biopsy (FNA) is usually applied to distinguish benign from malignant thyroid nodules. However, cytological analysis cannot always allow a proper diagnosis. We believe that the improvement of the diagnostic capability of pre-surgical FNA could avoid unnecessary thyroidectomy. In a previous study, we performed a proteome analysis to examine FNA collected after thyroidectomy. With the present study, we examined the applicability of these results on pre-surgical FNA. We collected pre-surgical FNA from 411 consecutive patients, and to obtain a correct comparison with our previous results, we processed only benign (n = 114), papillary classical variant (cPTC) (n = 34) and papillary tall cell variant (TcPTC) (n = 14) FNA. We evaluated levels of five proteins previously found up-regulated in thyroid cancer with respect to benign nodules. ELISA and western blot (WB) analysis were used to assay levels of L-lactate dehydrogenase B chain (LDHB), Ferritin heavy chain, Ferritin light chain, Annexin A1 (ANXA1), and Moesin in FNA. ELISA assays and WB analysis confirmed the increase of LDHB, Moesin, and ANXA1 in pre-surgical FNA of thyroid papillary cancer. Sensitivity and specificity of ANXA1 were respectively 87 and 94% for cPTC, 85 and 100% for TcPTC. In conclusion, a proteomic analysis of FNA from patients with thyroid nodules may help to distinguish benign versus malignant thyroid nodules. Moreover, ANXA1 appears to be an ideal candidate given the high sensitivity and specificity obtained from ROC curve analysis.

## Introduction

Nodular thyroid disease is a frequent finding in clinical practice and it is diagnosed in 4 to 7% of the adult population [1]. However, the incidence of thyroid cancer is low and most thyroid nodules are benign. The annual incidence of thyroid cancer in areas not affected by nuclear fallout has been reported to range between 1.2 and 2.6 cases per 100,000 in men and between 2.0 and 3.8 cases per 100,000 in women, with higher incidences in countries, such as Sweden, France, Japan, and the United States [2], [3].

Ultrasound guided fine-needle aspiration (FNA) cytology is a safe and sensitive diagnostic procedure in the management of thyroid lesions. The application of proteomics to pre-surgical FNA could improve the diagnosis of thyroid nodules reducing unnecessary thyroidectomies with a positive impact on health care costs and patient treatment. The identification and validation of a diagnostic proteomic biomarker panel would be very helpful in distinguishing benign from malignant nodules.

Until now many studies have been carried out in thyroid cancer research, and over the last years, besides genetic studies, proteomics has made headway in discovering potential thyroid biomarkers. Proteins are excellent targets in disease diagnosis and can be extracted from serum [4], tissues [5]–[7], and cell cultures [8], [9] for analysis with different techniques.

In a previous study, we performed a comparative proteome analysis to examine FNA collected immediately after total thyroidectomy [10]. We investigated the global changes of FNA protein patterns of two variants of malignant papillary thyroid cancer (PTC): the classical variant PTC (cPTC) and the tall cell variant PTC (TcPTC). Differences in protein expression between tumor and non-tumor tissue were identified using two-dimensional electrophoresis (2DE) and matrix-assisted laser desorption ionization time-of-flight/mass spectrometry (MALDI-TOF/MS) [10]. Our study allowed us to highlight proteins which were useful to give insights on molecular mechanisms of thyroid tumours and for this reason, we considered it was worth transferring this information on the less invasive pre-surgical FNA. Therefore, with the present study, we aimed to examine the applicability of results obtained on the surgical specimens on a larger number of pre-surgical thyroid FNA samples. Hence, we evaluated the levels of five proteins, previously found up-regulated in cancer with respect to controls, by enzyme-linked immunosorbent assay (ELISA) kit and western blot analysis (WB). We decided to test proteins with different functional properties, considering both the fold variation and the *p*-value in each functional class that we found in our previous study. These proteins were L-lactate dehydrogenase B chain (LDHB); Ferritin heavy chain (FHC); Ferritin light chain (FLC); Annexin A1 (ANXA1), and Moesin [10].

## Materials and Methods

### Study Design

This study adopted a phased approach. In the first part we collected pre-surgical FNAs from 411 consecutive patients. After their histological examination, we selected 162 samples from this large cohort of patients because we processed only benign, cPTC, and TcPTC FNAs. This choice was made to obtain a correct comparison with the results described in our previous work for surgical specimens. In the second phase we performed WB and ELISA to evaluate in these samples the levels of five proteins that we found up-regulated in cancer respect to control in our previous work. Our interest was to examine, on the pre-surgical thyroid FNAs, the applicability of results obtained on the surgical specimens. Finally, since ROC curves pointed out the ability of ANXA1 in separating malignant and benign groups, we investigated the levels of this protein in a subset of FNAs from patients with follicular lesion of undetermined significance.

### Patients

Ultrasound-guided FNA was performed as a part of the standard diagnostic protocol for patients with thyroid nodules in the Department of Endocrinology at the University of Pisa in Italy. Great care was given to collect only material from nodular lesions with the help of ultrasound. After the aspirate was smeared for conventional cytology, the leftover material in the needle was washed with 300 µl of saline solution and the liquid centrifuged at 2300*xg* for 10 min at 4°C. The supernatants were stored at −80°C until use.

A specimen was considered as satisfactory if there were six groups of epithelial cells with at least 10 cells per group. According to FNA analysis, the nodules were classified as benign, follicular lesions of undetermined significance (FLUS) (high to moderate cellularity and the presence of microfollicular pattern of growth with or without Hurthle cell change and scant colloid), suspicious for malignancy or malignant, and non-diagnostic or inadequate (due to limited cellularity or poor preservation and fixation), following the guidelines of National Cancer Institute thyroid fine needle aspiration state of the science conference [11].

We collected FNA samples from 411 consecutive patients, without considering clinical or other parameters. Cytological evaluation classified these FNAs in benign (n = 254), FLUS (n = 76), malignant (n = 25), suspicious for malignancy (n = 21) or non diagnostic (n = 35). All the patients with benign thyroid nodules were followed conservatively for at least 5 years by annual ultrasound examination. The patients with malignant or suspicious for malignancy or FLUS underwent thyroid surgery after completion of the clinical and cytological evaluation. The histological examination, after surgical intervention, completed the diagnosis for these samples. To obtain a correct comparison with the results described in our previous work for surgical specimens [10], we processed only benign (n = 114), cPTC (n = 34), and TcPTC (n = 14) FNA samples found in our cohort of patients. These PTC have had a cytological evaluation as benign (n = 114), malignant (n = 24), suspicious for malignancy (n = 20) and FLUS (n = 4). Moreover, we selected 24 FNAs from patients with FLUS (cPTC n = 4, follicular carcinoma n = 17, trabecular carcinoma n = 3) for the further validation of ANXA1.

Serum thyroxin (FT4), thriiodothyronine (FT3) and thyrotropin (TSH) values were in the normal range in all patients. No serum antithyreoglobuline (TgAb) and antithyreoperoxidase (TPOAb) antibodies were detectable. Serum calcitonin was undetectable in all patients.

#### Laboratory evaluation of thyroid function

Serum free FT4 and free FT3 were measured with a chemiluminescent method (Vitro System, Ortho-Clinical Diagnostics, Rochester, NY, USA). TSH was assessed by ultrasensitive commercial chemiluminescent method (Immulite 2000; Diagnostic Products, Los Angeles, CA, USA). TPOAb and TgAb were measured using a two-step immunoenzymatic assay (AIA-Pack TgAb and TPOAb; Tosoh, Tokyo, Japan). Serum calcitonin was measured by immunoradiometric assay (CisBio International, Gif-sur-Yvette, France).

#### Ethics statements

This study was approved by the Local Ethics Committee (Comitato per la Sperimentazione Clinica dei Farmaci, Azienda Ospedaliera Universitaria Pisana; reference number 3016) and signed consent forms were obtained from all patients.

### ELISA Assay

The levels of LDHB, FHC, FLC and Moesin were detected by enzyme-linked immunosorbent assay (ELISA) kits (*Uscn* life science Inc.) according to the manufacturer’s instructions. The Lower Limit of Detection of these assays was less than 1.14 ng/ml; 5.5 pg/ml; 14.7 pg/ml l; and 0.059 ng/ml for LDHB, FHC, FLC, and Moesin respectively.

### Western Blotting

WB analysis was performed to estimate ANXA1 levels in FNA. 10 µg of proteins were resolved by 12% SDS-PAGE gels and transferred onto nitrocellulose membranes (0.2 µm) using a voltage of 100 V for 30 min (Criterion Blotter, Biorad, CA, USA). Non-specific binding was prevented by blocking the membranes with 3% low fat dried milk, 0.2% (v/v) Tween 20 in PBS (10 mM NaH_2_PO_4_, pH 7.4, 0.9% NaCl) (PBS/milk) for 1 h at room temperature. After their blocking, the membranes were incubated overnight at 4°C in PBS/milk containing primary antibody ANXA1 (rabbit monoclonal, 1∶1000 dilution; Cell Signaling Technology, MA, USA). HRP-conjugated goat anti-rabbit (1∶10000 dilution; Stressgen, NY, USA) was used as a secondary antibody. Immunoblots were developed using the ECL detection system (PerkinElmer, MA, USA). The chemiluminescent images were acquired by LAS4010 (GE Health Care, WI, USA). For the comparison of protein expression levels, the antigen-specific bands were quantified using the Image Quant-L (GE Health Care, WI, USA).

### Statistical Analysis

Results were expressed as mean ± SEM. Differences between groups were determined using Student’s *t* test. Data were considered statistically significant at *p-value <*0.05. Receiver operating characteristic (ROC) curve was performed to assess sensitivity and specificity (MedCalc 9.6.4.0 software).

## Results

### Validation of Differentially Expressed Proteins by ELISA and WB Analysis

**Figure 1:**
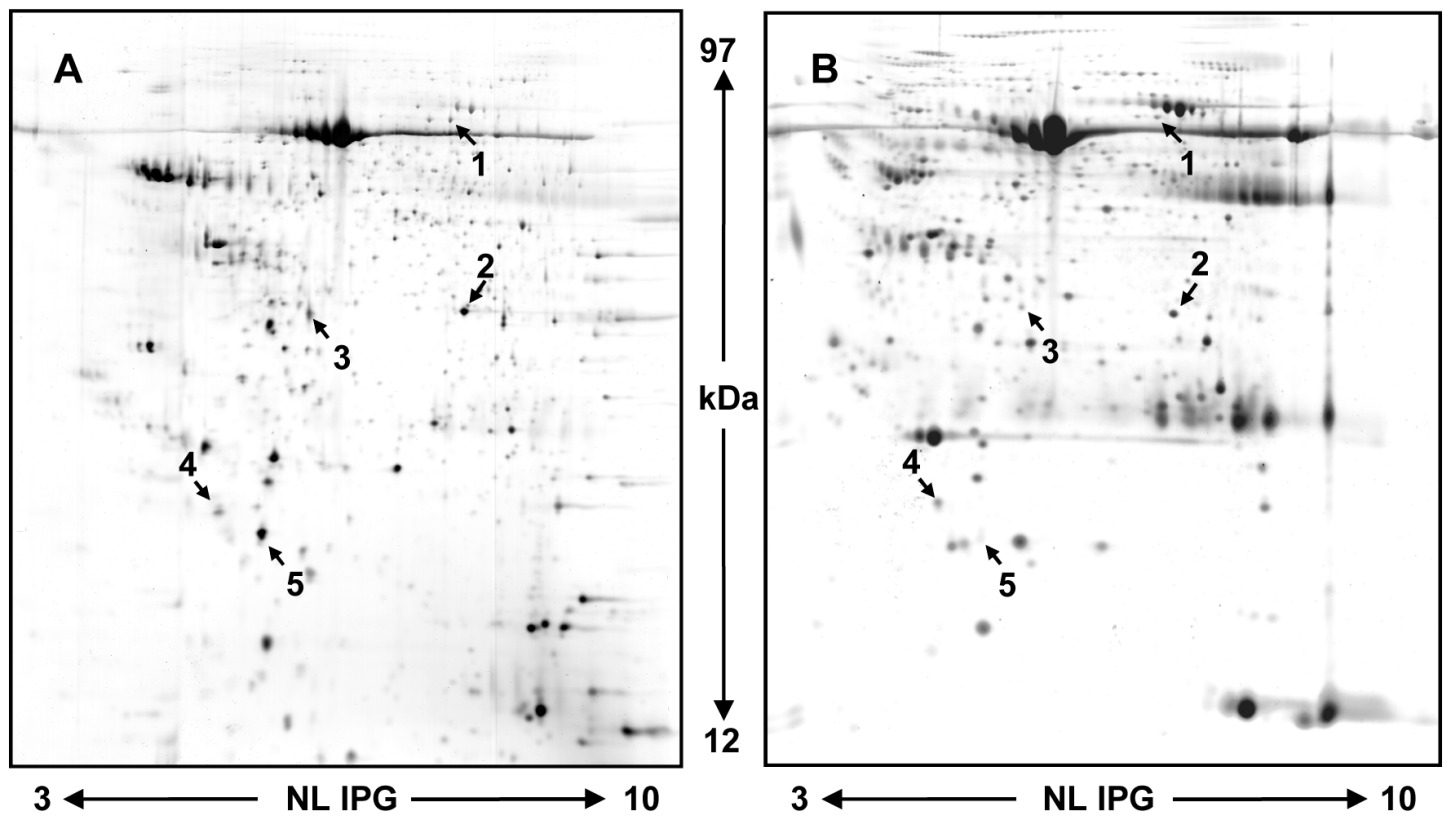
2DE patterns of human thyroid FNA. Post-surgical TcPTC (A) and pre-surgical (B) TcPTC FNA. A total of 250 µg of proteins was separated by 2DE using 18 cm pH 3–10NL strips and 12.5% SDS-PAGE (see Methods S1). The five proteins whose levels were evaluated by ELISA kit and WB are indicated. 1 moesin, 2 ANXA1, 3 LDHB, 4 FHC and 5 FLC.

In order to verify the analogy of pre- and post- surgical thyroid FNA samples we compared their proteome profile (Figure 1 A and B). Pre-surgical FNA samples were pooled according to their different classes (benign, cPTC, TcPTC) and subjected to 2DE (see Methods S1). Gel images were analysed with the Progenesis Same Spot v4.1 (Nonlinear Dynamics) software [12]. This software includes the SpotCheck function. SpotCheck is a separate quality control (QC) workflow within Progenesis Same Spot v4.1 which allowed us to assess objectively the quality of our gels images. For each class of gels (benign, cPTC, and TcPTC) we selected a gold standard one (a reference gel) and each gel was tested against it. Therefore we compared pre- against post-surgical FNA and we obtained coefficients of variation (CV) ranging from 91.9 to 85% confirming a strong analogy between pre- and post-surgical FNA samples. Moreover, by using the software, we were able to locate our five protein spots in the gel image of pre-surgical FNA. These spots were cut out from the gel and identified by NanoLC-ESI-MS/MS analysis (see Methods S1). Thus, we confirmed the identification of our five selected proteins.

**Figure 2:**
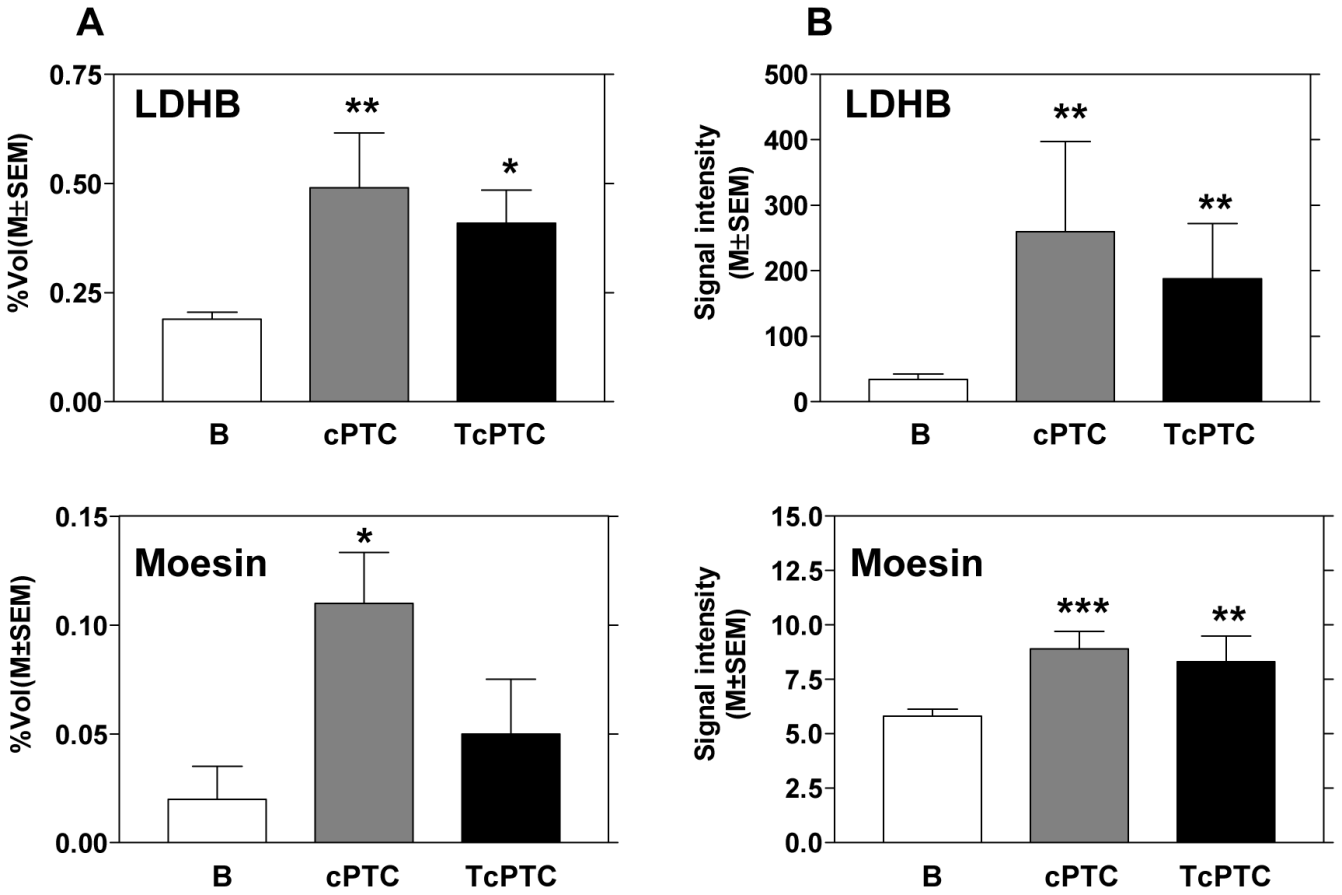
Expression of LDHB and Moesin in post-surgical and pre-surgical FNA. (A) Histograms of the percentage volumes previously obtained in FNA of surgical specimens with 2DE for LDHB and Moesin; (B) The ELISA array detection results for LDHB and Moesin in pre-surgical FNA. Each bar represents the mean ± SEM of percentage volume and signal intensity. Analysis B vs cPTC, B vs TcPTC (*p<0.05, **p<0.01, ***p<0.001).

Then, using ELISA assay or WB analysis, the different expression of five proteins of interest was validated. Figure 2, panel A shows the results previously obtained by 2DE analysis for LDHB and moesin in post-surgical FNA. Panel B shows the ELISA array detection results for LDHB and moesin in pre-surgical FNA. With ELISA assay we confirmed the significant increase of LDHB found with 2DE in both two variants of malignant PTC respect to benign controls (B), (p = 0.0078 B vs cPTC, and p = 0.0012 B vs TcPTC) (table 1). Moesin resulted up-regulated only in cPTC after 2DE analysis, but the validation with ELISA in a larger number of samples highlighted also an increase in TcPTC compared to controls (table 1). With regard to ferritins, the significant increase of expression for FHC (p = 0.011) and FLC (p = 0.015) was confirmed in pre-surgical FNA of cPTC variant, but not in TcPTC.

**Table 1:** Results of percentage volume, signal intensity and optical density of proteins studied.

	2DE					ELISA				
	%vol (M±SEM)			*p-value*		signal intensity (M±SEM)			*p-value*	
	B	cPTC	TcPTC	B*vs*cPTC	B*vs*TcPTC	B	cPTC	TcPTC	B*vs*cPTC	B*vs*TcPTC
**LDHB**	0.19±0.02	0.49±0.13	0.41±0.1	0.0041	0.0167	34±8.7	260±138	188±85	0.0078	0.0012
**Moesin**	0.02±0.02	0.11±0.02	0.05±0.03	0.0175	0.1636	5.8±0.32	8.9±0.79	8.3±1.18	<0.001	0.006
**FLC**	0.005±0.003	0.16±0.05	3.58±1.02	<0.001	<0.001	27911±9447	58801±16328	7379±840	0.015	0.8
**FHC**	n.d.	n.d.	2.69±1.2	n.d.	<0.001	765±100	1388±286	760±102	0.011	0.9
						**WB**				
						**optical density (M±SEM)**				
**ANXA1**	0.06±0.02	0.84±0.16	0.37±0.15	<0.001	0.0061	3.2e^+04^±1.7e^+04^	6.2e^+06^±1.7e^+06^	4.2e^+06^±1.4e^+06^	<0.001	<0.001

n.d. = not detectable.

**Figure 3:**
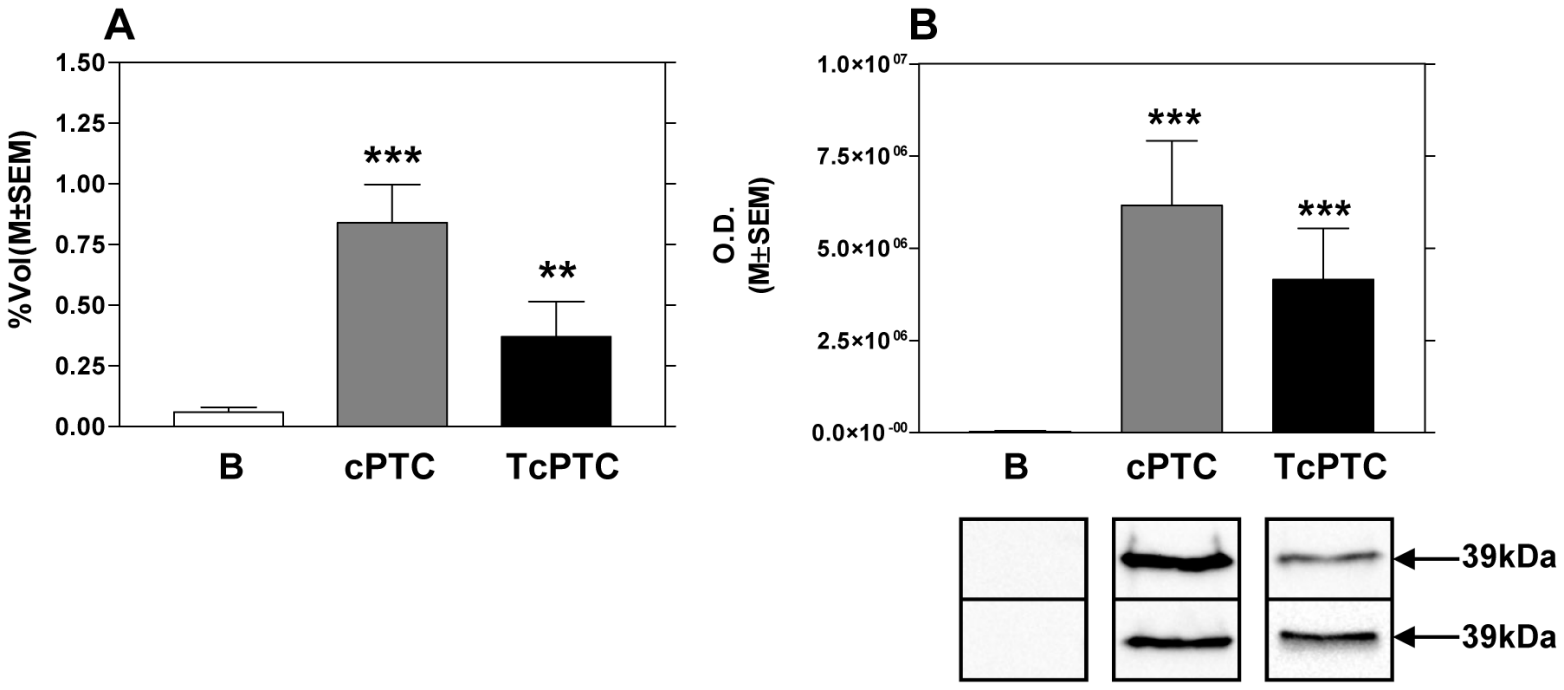
Expression of ANXA1 in post-surgical and pre-surgical FNA. (A) Histograms of the percentage volumes previously obtained in FNA of surgical specimens with 2DE for ANXA1; (B) Western blot analysis of ANXA1 in pre-surgical FNA. Each bar represents the mean ± SEM of percentage volume and optical density. Analysis B vs cPTC, B vs TcPTC (*p<0.05, **p<0.01, ***p<0.001).

Finally, to obtain a specific validation of ANXA1 in pre-surgical FNA samples and to eliminate potential cross-reactions in ELISA kit due to the high homology of members of annexin family, we carried out WB analysis using an antibody directed against a synthetic peptide corresponding to residues surrounding Val236 of human ANXA1 protein. WB pointed out the significant up-regulation of ANXA1 in cPTC and TcPTC, showing almost the absence of this protein in controls (Figure 3).

### ROC Curves

ROC curves were calculated to evaluate the ability of selected proteins to separate malignant and benign groups. For LDHB sensitivity and specificity were respectively 78 and 59% for cPTC, and 46 and 94% for TcPTC. Moesin showed a sensitivity of 64 and 79% and a specificity of 80 and 54% for cPTC and TcPTC respectively. Sensitivity was 95 and 55%, and specificity 47 and 82% for FHC and FLC, respectively, in cPTC, while in TcPTC the significant increase of ferritins was not confirmed.

**Figure 4:**
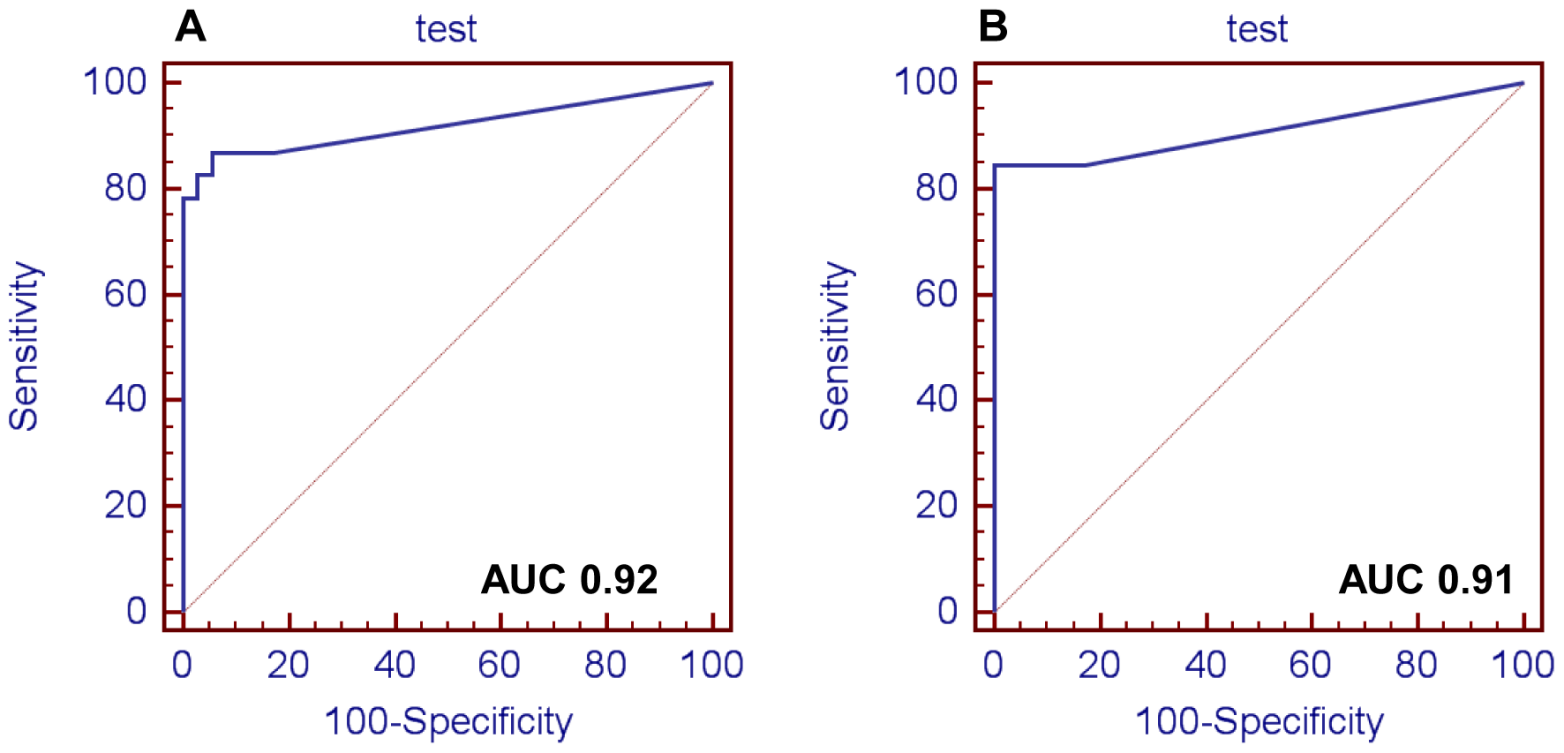
ROC curve of ANXA1. (A) B vs cPTC; (B) B vs TcPTC. AUC, area under the curve.

Finally, ANXA1 showed better results compared to the above mentioned proteins (Figure 4). The sensitivity and the specificity of the ANXA1 were respectively, 87 and 94.3% for cPTC and 84.6 and 100% for TcPTC with a criterion value of 1,09e^+05^ (cPTC) and 5,07e^+05^(TcPTC). The negative predictive value (NPV) was 0.92 and 0.95 for cPTC and TcPTC respectively, whereas the positive predictive value (PPV) was 0.91 and 1 for cPTC and TcPTC respectively (Figure 4).

### Expression of ANXA1 in FNAs Suspicious for Follicular Neoplasm and Suspicious for Malignancy

**Figure 5:**
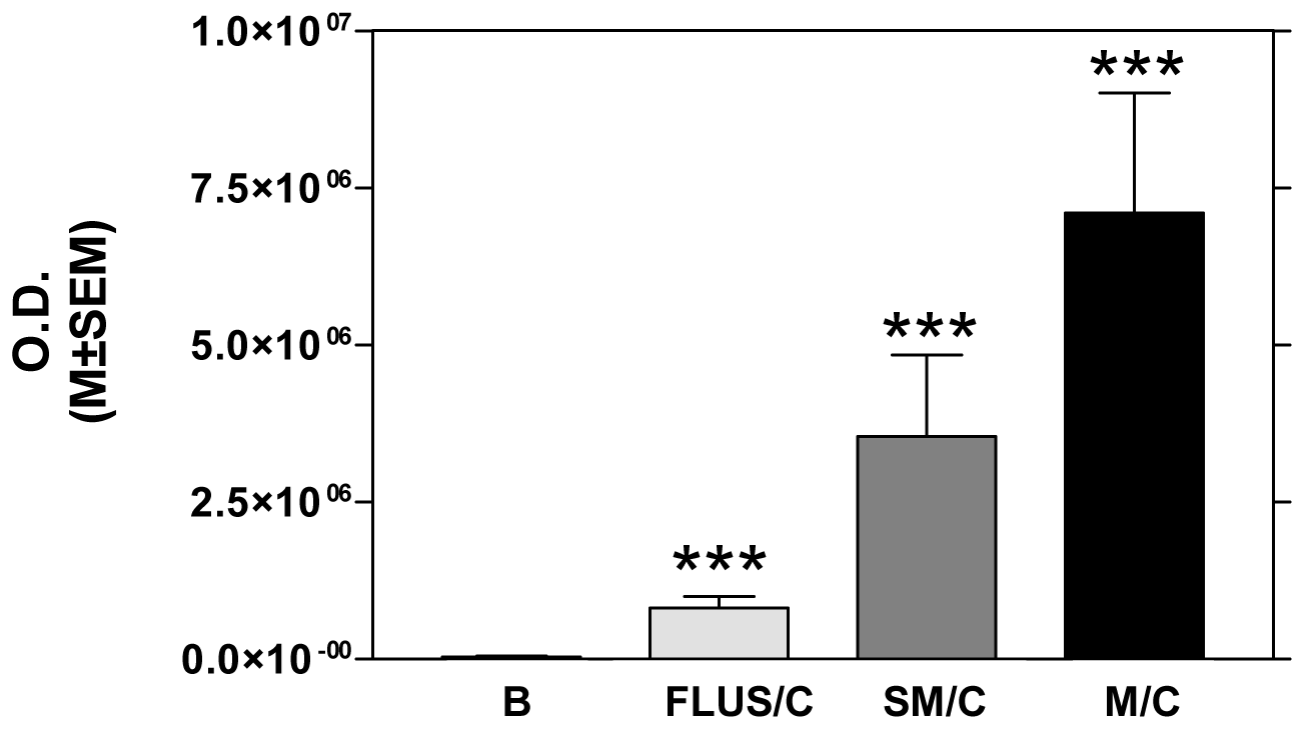
Expression of ANXA1 in FNAs suspicious for follicular neoplasm, suspicious for malignancy and malignant. The levels of ANXA1 were evaluated by WB analysis in a subset of FNAs suspicious for follicular neoplasm and suspicious for malignancy (SM). Samples were separated according to cytological and histological examination: FLUS with histological diagnosis of cancer (FLUS/C), samples SM with histological diagnosis of cancer (SM/C) and malignant samples confirmed cancer after histological examination (M/C). Each bar represents the mean ± SEM of optical density. Analysis B vs FLUS/C, B vs SM/C, B vs M/C (*p<0.05, **p<0.01, ***p<0.001).

The levels of ANXA1 were evaluated by WB analysis in a subset of FNAs suspicious for follicular neoplasm and suspicious for malignancy. As depicted in figure 5, samples were separated according to cytological and histological examination. Our results pointed out the significant up-regulation of ANXA1 in all type of cancer respect to control.

## Discussion

In our previous work we studied PTC samples using 2DE and MALDI-TOF/MS. The research was carried out on 13 samples of FNA collected after total thyroidectomy and in which we found changes in protein expression between cPTC, TcPTC, and controls. Our results demonstrated that FNA may be used in the comparative analysis of thyroid tumours [10]. In the present work we tested the possibility to transfer these findings also on pre-surgical FNA, since it is usually applied for the detection of thyroid cancer. With this purpose, first we compared 2DE patterns of pre-surgical and post-surgical samples. Then, in a larger number of pre-surgical FNA samples, we investigated the expression of some proteins which had been found significantly up-regulated in PTC in the previous proteomic study. We chose five proteins with different functional properties: LDHB (metabolism), FHC and FLC (storage), ANXA1 (apoptosis) and moesin (structural). We examined ANXA1 expression by WB while ELISA assay was used for LDHB, FHC, FLC and moesin.

Overall, our results demonstrated the similarity of proteomic profile of pre-surgical FNA and FNA made on surgical specimens; moreover, the previous potential biomarkers were confirmed, suggesting their applicability on the diagnosis of thyroid cancer. In particular, the alteration of LDHB enzyme expression is widely documented [13]–[16]. In fact, it is widely accepted that cancer cells proliferation is characterized by fermentation of glucose to lactate [17]. This is the Warburg effect, which is considered as one of the key features of tumorigenesis and which promotes rapid uncontrolled proliferation, but also confers invasive property [18], [19]. We found in PTC a significant up-regulation of LDHB, and in both studies this increase was greater in cPTC than in TcPTC (Fig. 2 A and B). The same result was obtained with a structural protein: moesin. Moesin belongs to the ERM (ezrin, radixin and moesin) family that takes part in many signalling pathways playing a crucial role in cell morphology, adhesion and motility [20]–[22]. Moreover, they have been shown to be involved in carcinogenesis [23]–[26]. It has been recently proposed that moesin contributes to cancer cell proliferation, migration, and invasion in cancer [27]. In addition to our previous study [10], other authors found moesin between the differentially expressed proteins in thyroid tumours [28], [29], and our ELISA assay confirmed the significant up-regulation of this protein not only in cPTC, but also in TcPTC.

The results obtained with WB of ANXA1 are still in agreement with those we obtained by 2DE, and they add to many data in literature that support a possible role of annexin family in the oncogenic process. In fact, annexins are the most common proteins in cancers revealed by proteomic and protein functional studies [30]. As calcium- and phospholipid-binding proteins, annexins are associated with the lymph node metastasis of hepatocarcinoma, lung cancer, colon cancer, head and neck cancer, nasopharyngeal cancer, and oesophageal and oesophagogastric junction adenocarcinoma [30]. With regard to thyroid carcinoma, previous proteomic studies revealed increased levels of annexin A2, A5, A1 [6], [31]–[33]. In our case in post- and pre-surgical FNA we also found the up-regulation of ANXA1 [10], a 39 kDa protein that may promote apoptosis [34]. Moreover, it has been recently proposed that ANXA1 enhances breast cancer invasion, at least in part, through the activation of NF-kappaB and the expression of the matrix metalloproteinase -9 gene [35].

Besides this validation of previous results in PTC, we performed a further evaluation of the levels of ANXA1 in a subset of pre-surgical FNAs suspicious for follicular neoplasm and suspicious for malignancy that had a diagnosis of cancer after histological examination. We found a significant up-regulation of this protein for each type of cancer, supporting the relevance of ANXA1 as ideal candidate biomarker in the diagnosis of thyroid cancer.

Finally, the validation with ELISA assay confirms our previous findings on ferritins but only in cPTC respect to controls, and not in TcPTC variants. Therefore, ferritins have not been validated as a malignant tumour marker in pre-surgical TcPTC FNA. Ferritin is a protein involved in iron storage, consisting of 24 H (heavy) and L (light) subunits, encoded by distinct genes [36]. Abnormal ferritin expression has been observed in a large number of tumour types, and it has been suggested that it may act as a tumour marker in neuroblastoma and other cancers, such as hepatic cancer, lung cancer, leukemia in remission, and breast cancer recurrence or metastasis [37], [38]. Thus, not only is ferritin considered an iron storage indicator, but also a malignant tumour marker, even if recently Kim et al. have found that ferritin is not associated with the risk of cancer [39]. In our previous study we found an increment of FHC and FLC in TcPTC which has not been confirmed. Even Deshpande and co-workers did not observe a significant difference in serum ferritin concentration in thyroid carcinoma patients as compared to controls, but it appeared to be influenced by the presence of metastasis and the type of histology [40].

In conclusion, having found in the FNA in vivo, and in a larger number of patients, significant differences in expression of proteins previously identified in the post-surgical FNA has confirmed the relevance of these putative biomarkers. Moreover, taken together, our studies indicate that thyroid FNA is feasible as a potential source of diagnostic biomarkers for thyroid cancer, and therefore, there is a clear need to carry out further investigations on other proteins previously found with 2DE analysis. The goal will be to identify combined biomarkers that could have potential for clinical use. In particular, the results obtained with ANXA1 in FLUS are encouraging for our future research, which is directed at studying follicular lesions, since their correct surgical management is really controversial.

## Supporting Information

Methods S1 Two-dimensional electrophoresis and NanoLC-ESI-MS/MS Analysis by LTQ-Orbitrap Velos analysis.
(DOCX)
