# Supplementary material for: Presence in the Pre-Surgical Fine-Needle Aspiration of Potential Thyroid Biomarkers Previously Identified in the Post-Surgical One
Source: PLoS One. 2013 Sep 2;8(9):e72911. doi: 10.1371/journal.pone.0072911 (PMC3759451; doi:10.1371/journal.pone.0072911)
Supplement: Methods S1 — Two-dimensional electrophoresis and NanoLC-ESI-MS/MS Analysis by LTQ-Orbitrap Velos analysis. (DOCX) [file pone.0072911.s001.docx]

**Methods S1**

**Two-dimensional electrophoresis**

250 μg of proteins, for each sample, were filled up to 400 µl in rehydration solution. Immobiline Dry-Strips (GE Healthcare; 18 cm, NL gradient pH 3-10) were rehydrated overnight in the sample then transferred to the Ettan IPGphor Cup Loading Manifold (GE Healthcare) for isoelectrofocusing (IEF). IEF was performed at 16°C and the proteins were focused for up to 70000Vh. The second dimension (SDS-PAGE) was carried out by transferring the proteins to 12.5% polyacrylamide gel, running at 16 mA/gel and 10°C for about 16 h. The gels were stained with Ruthenium II tris (bathophenanthroline disulfonate) tetrasodium salt (SunaTech Inc.) (RuBP). After electrophoresis, the gels were fixed in 1% phosphoric acid (v/v) and 30% ethanol for 1h then were stained overnight with 1 mM RuBP in 1% phosphoric acid and 30% ethanol. After this time the gels were destained for 5 hours in 1% phosphoric acid and 30% ethanol and rinsed in water prior to acquisition by “ImageQuant LAS4010” (GE Health Care). The analysis of images was performed using Progenesis Same Spot (Nonlinear Dynamics) software. This software generates 2DE analyses which are robust and accurate. The gels were aligned to place all spots in exactly the same location, and then spot detection produced a complete data set since all gels contain the same number of spots, each matched to its corresponding spot on all gels. The 2DE experiments were performed in triplicate.

**NanoLC-ESI-MS/MS Analysis by LTQ-Orbitrap Velos analysis.**

Gels pieces were destained in 100 % EtOH during 2 hours. Subsequently, gel pieces were rehydrated with 100 μl of 50 mM ammonium bicarbonate for 15 min and dehydrated with 100 μl of 50 mM ammonium bicarbonate in 30% AcN for 15 min.

Gel pieces were then dried for 30 minutes in a Centrivap vacuum centrifuge (Labconco, Kansas City, USA). Dried pieces of gel were rehydrated for 45 min at 4°C in 20 μl of trypsin porcine (Sigma) solution (6.25 ng/μl in 50 mM ammonium bicarbonate) and incubated at 37°C overnight. Extraction of the peptides was performed with 20 μl of 1% trifluoroacetic acid (TFA) for 30 min at room temperature with occasional shaking. The TFA solution containing the proteins was transferred to a polypropylene tube. A second extraction of the peptides was performed with 20 μl of 0.1% TFA in 50% AcN for 30 min at room temperature with occasional shaking. The second TFA solution was pooled with the first one. The volume of the pooled extracts were dried completely and finally resuspended in CH3CN/FA 50 %/0.1%.

LC-ESI-MS/MS was performed on a linear trap quadrupole (LTQ) Orbitrap Velos from Thermo Electron (San Jose, CA, USA) equipped with a NanoAcquity system from Waters. Peptides were trapped on a home-made 5 µm 200 Å Magic C18 AQ (Michrom) 0.1 × 20 mm pre-column and separated on a home-made 5 µm 100 Å Magic C18 AQ (Michrom) 0.75 × 150 mm column with a gravity-pulled emitter. The analytical separation was run for 23 min using a gradient of H2O/FA 99.9 %/0.1% (solvent A) and CH3CN/FA 99.9 %/0.1% (solvent B). The gradient was run as follows: 0–5 min 95 % A and 5 % B, then to 65 % A and 35 % B at 6 min, and 20 % A and 80 % B at 7 min at a flow rate of 220 nL/min. For MS survey scans, the orbitrap (OT) resolution was set to 60000 and the ion population was set to 5 × 105 with an m/z window from 400 to 2000. For protein identification, up to five precursor ions were selected for collision-induced dissociation (CID). For MS/MS in the LTQ, the ion population was set to 1 × 10e4 (isolation width of 2 m/z) while for MS/MS detection in the OT, it was set to 1 × 10e5 (isolation width of 2 m/z). The normalized collision energies were set to 35% for CID.

**Protein identification**

Peak lists were generated from raw orbitrap data using the embedded software from the instrument vendor (extract_MSN.exe). The monoisotopic masses of the selected precursor ions were corrected using an in-house written Perl script (Scherl et al., Proteomics 2008, 8, p. 2791). The peaklist files were searched against the UniProtKB/Swiss-Prot database (Release-2011_08 of 21-Sep-2011) using Mascot (Matrix Sciences, London, UK). Human taxonomy (20323 sequences) was specified for database searching. The parent ion tolerance was set to 10 ppm. Variable amino acid modifications were oxidized methionine and fixed amino acid modifications were carbamidomethyl cysteins. Trypsin was selected as the enzyme, with one potential missed cleavage, and the normal cleavage mode was used. The mascot search was validated using Scaffold 3.6.0 (Proteome Software, Portland, OR). Only proteins matching with two different peptides with a minimum probability score of 95% were considered identified.
